# Enhancing the understanding of association between breast-conserving surgery with a lower incidence of suicide among females with breast cancer

**DOI:** 10.1097/JS9.0000000000001530

**Published:** 2024-05-03

**Authors:** Zujun Wen

**Affiliations:** Department of Pharmacy, Heyuan People’s Hospital, Heyuan, People’s Republic of China

*Dear Editor,*

I read with great interest the retrospective cohort study by Guo *et al*.^1^. Breast-conserving surgery (BCS) is associated with a lower incidence of suicide among females with breast cancer in the United States: a population-based retrospective cohort study. This study, which contained information from over a million US female breast cancer patients, discovered that BCS was associated with a suicide death risk. The authors have commendably showcased how BCS was linked to a significantly decreased suicide rate among female breast cancer patients, demonstrating its impressive methodology and insightful information. The primary findings, highlighting BCS presents a strong alternative for raising cancer patients’ self-esteem and quality of life while also offering a fresh viewpoint on cancer treatment, which are highly significant and timely. Although the study’s meticulous efforts and noteworthy achievements are greatly acknowledged, several valuable suggestions are offered for further development.

First, the choice of statistical model. Notwithstanding its widespread acceptance, the Cox regression model may overestimate in situations when there are conflicting hazards. It’s possible that traditional survival analysis techniques fail to completely take into consideration how additional events affect the main result. A competing risk model or the difference in restricted mean survival time (dRMST) test might offer a more thorough viewpoint^2,3^.

Second, the potential covariates. It makes sense that the interplay between age, race, year of diagnosis, residential area, median household income, stage, follow-up time, surgical modality, and cause of death should be taken into account in the study. However, taking into account additional plausible variables including marital status^4^, physical diseases (e.g. gastrointestinal disease^5^, etc.), emotional support, and social attention^6,7^ could potentially strengthen the validity of the results.

Third, performing sensitivity analyses with the exclusion of certain populations, such as individuals with depressive syndromes, especially major depression, might help to better understand the relationship, as depression markedly increased the probability of suicide death in cancer patients^8^.

In conclusion, the research conducted by Guo G *et al*. represents a noteworthy advancement in our comprehension of the variations in the suicide rate among breast cancer survivors between BCS and mastectomy. The current study’s findings offer a fresh viewpoint on cancer care while highlighting the significance of raising the quality of life and self-esteem of female breast cancer patients. Improved treatment and preventative approaches may result from such studies, which might help a great deal of people. My suggestions merely serve to enhance a superb piece of research. I hope to read more thoughtful works from the writers in the future.

## Ethical approval

Not applicable.

## Consent

Not applicable.

## Sources of funding

I received no financial support to produce this manuscript.

## Author contribution

Z.W.: contributed all the content of the current manuscript.

## Conflicts of interest disclosure

I declare that there are no conflicts of interest.

## Research registration unique identifying number (UIN)

Not applicable.

## Guarantor

Zujun Wen.

## Data availability statement

Not applicable.

## Provenance and peer review

Not applicable.
